# The hidden network: community sense, social desirability, and their protective influence on negative emotions in aging populations

**DOI:** 10.3389/fpubh.2025.1572044

**Published:** 2025-04-23

**Authors:** Ying Chu, Yanhong Liu, Xiaotong Qu, Xiaoxiang Wang

**Affiliations:** ^1^Department of Marxism Sinicization, School of Marxism, Liaoning Normal University, Dalian, China; ^2^Department of Nursing, The First Affiliated Hospital of Dalian Medical University, Dalian, China

**Keywords:** social capital, social support, community sense, social desirability, negative emotions, older adult

## Abstract

**Background:**

With aging populations, understanding mechanisms linking social factors to emotional well-being in the older adult is critical. This study examines how social capital, social support, community sense, and social desirability interact to influence negative emotions.

**Methods:**

A cross-sectional survey of 904 older adult individuals in Liaoning, China, utilized standardized scales: GDS-10 (negative emotions), SSRS (social support), BSCS (community sense), and MCSDS (social desirability). Mediation and moderation analyses were conducted.

**Results:**

The results demonstrated significant negative correlations between social capital/social support and negative emotions. Social support was identified as a mediating factor linking social capital to reduced negative emotions. Additionally, community sense and social desirability moderated the relationship between social support and negative emotions, with stronger community belonging and higher social desirability amplifying the protective effect of social support.

**Conclusion:**

These findings underscore the importance of fostering social capital, strengthening social support networks, cultivating community integration, and addressing social desirability biases to alleviate negative emotions in the older adult. The study provides actionable insights for designing targeted mental health interventions aimed at improving emotional well-being in aging populations.

## Highlights

- Social support mediates the relationship between social capital and negative emotions in the older adult.
- Community sense and social desirability jointly moderate the effect of social support on alleviating negative emotions.
- This study is the first to explore the synergistic moderating effect of community sense and social desirability on negative emotions in older adult populations.

## 1 Introduction

### 1.1 Research background

As a result of the rapid acceleration of global population aging, mental health problems among the older adult, especially negative emotions, have become a hot issue in the field of public health. According to the World Health Organization (WHO), negative emotions affect between 10 to 15 percent of the world’s older adult population (1). As China continues its economic development and medical conditions improve, the proportion of older adult population is rapidly increasing, and is expected to reach over 20% of the total population by 2030 (2). This trend not only brings challenges to the social security system but also has profound influence on the quality of life and mental health of the older adult (3).

The prevalence of negative emotions among the older adult varies considerably from one region to another and also from one culture to another. Negative emotions are different in urban and rural older adult populations in China, partly because of social support and social capital differences. Because of the lack of social resources and an undeveloped social support system, rural older adult people have a relatively higher prevalence of negative emotions, and urban older adult people, though they have a relatively better social support system, also suffer from the fast pace of modern life and the estrangement of social relationships (4).

### 1.2 Conceptual framework and review of the literature

#### 1.2.1 Social support and negative emotions

Social support, as an important social resource, according to social support theory, can reduce negative emotions when facing stress and difficulties, enhance psychological resilience, and reduce the occurrence of negative emotions (5). In fact, good social support has been associated with emotional comfort and, through informational and material support, helps the older adult better cope with life challenges (6). The social support system in China relies mainly on family and community, with traditional culture believing that family mutual support is an important source of social support (7).

However, traditional family support is weak with the change of social structure, and the community support system is not yet established, leaving social support lacking for the older adult, so the risk of negative emotions increases (8). The mechanism of such social support in negative emotions in the older adult is thus important for developing effective intervention measures.

#### 1.2.2 Social capital and negative emotions

According to social capital theory, social capital, which facilitates building and maintaining social networks, increases one’s access to social resources and is thus positively correlated with mental health (9). When older adult people experience high social capital, they have richer social interactions, feel a greater sense of community belonging, and have a lower frequency of negative emotions. Social capital directly affects the mental health of the older adult and affects them indirectly through intermediate variables such as social support proposed through this research (10).

The building of social capital in China is centered around family, sociable neighborhood relationships, and community organizations. In particular, senior individuals with high social capital tend to own more social resources and supportive networks such as emotional support, information exchange, and material aid, which can alleviate negative emotions (11). Moreover, social capital can enhance the self-efficacy and social participation level of older adult people, and subsequently their mental health status (12).

#### 1.2.3 Distinction between social capital and social support

While social capital and social support are related concepts, they represent distinct constructs in both theory and measurement. Social capital refers to the structural and cognitive resources embedded within social networks that facilitate collective action (13). It encompasses the breadth, quality, and potential value of one’s social connections. In contrast, social support represents the actual functional assistance received through these networks, including emotional comfort, informational guidance, and material aid (12, 14). Conceptually, social capital can be understood as the ‘infrastructure’ that enables social support to flow, while social support represents the actual ‘resources’ that are exchanged within these networks. This distinction explains our hypothesis that social support mediates between social capital and negative emotions.

#### 1.2.4 Community sense and community participation

Although community sense and community participation are indicators that measure a person’s sense of belonging and involvement in their community, participation in an active community has been linked to helping to foster a strong social support network and feeling part of the community, which can lead to a reduction in negative emotions (15). With the rapid development of urbanization in China, the form of community structure and the pattern of social interaction have changed greatly, and the sense of community and community participation are increasingly important (16).

Additionally, community participation provides rich social activities and interaction opportunities to promote the connections and mental health status of the older adult in the community (17). Thus, it has important significance for the mental health of the older adult population to study the influence of sense of community and community participation on negative emotions.

#### 1.2.5 Negative emotions and social desirability

Social desirability is an individual’s propensity to seek recognition and approval from others in their social interactions. High social desirability can reduce the appearance of negative emotions by raising the person’s self-esteem and social identity. But too much social desirability can lead individuals to shield their true feelings in stressful situations, making them more susceptible to showing negative emotions (18). In addition to the need for the recognition of others, social desirability in Chinese social culture also means that individuals achieve self-worth through social roles and social status. The social desirability might make the older adult feel important and valued in society, thereby reducing the incidence of negative emotions (19). However, excessive social desirability might lead some older adult individuals to hide their true feelings in pursuit of others’ approval, resulting in psychological distress and negative emotions (20). Thus, both the amplification and attenuation of negative emotions must be regulated in practice through the social desirability of humanistic nature.

#### 1.2.6 Interactive effects of community sense and social desirability

While previous sections have discussed community sense and social desirability separately, their interactive effects merit special attention. The relationship between social support and negative emotions cannot be fully understood by examining community sense or social desirability in isolation. Evidence suggests these constructs work synergistically rather than independently (21). Community attachment alone may be insufficient to buffer negative emotions without corresponding alignment with social norms (21). Similarly, conformity to social expectations provides limited protection against negative emotions without meaningful community integration (22).

This interactive perspective is particularly relevant in the Chinese cultural context, where collectivist values emphasize both community belonging and social harmony (23). The older adult in Chinese society face unique challenges as traditional family structures evolve and community dynamics change, making the interplay between objective social bonds and subjective social identity especially influential in shaping emotional experiences (24). By examining their interactive effect, we capture the dialectical relationship between objective social bonds and subjective social identity that shapes emotional resilience in aging populations, extending beyond what either factor could explain independently.

#### 1.2.7 Marxist theory and psychosocial factors in aging

From a Marxist theoretical perspective, the psychosocial factors examined in this study reflect the dialectical relationship between social being and social consciousness. Social capital represents the material social relations that older adult individuals have accumulated throughout their lives, while social support functions as the practical manifestation of these relations. Community sense embodies what Marx termed ‘species-being’ - the connection between individuals and their broader social environment (25). Social desirability, meanwhile, represents the internalization of social consciousness that guides behavior within collective contexts (26).

### 1.3 Research problem

Psychosocial factors su**c**h as social capital, social support, community sense, and social desirability are thought to have important roles in the generation and development of negative emotions in the older adult population. The purpose of this study is to examine the relationships between these psychosocial factors and negative emotions in the older adult and to examine the moderating roles of community sense and social desirability in these relationships. Specifically, the research hypotheses are as follows:

- Hypothesis 1: Negative emotions of the older adult are significantly negatively correlated with social capital.
- Hypothesis 2: The relationship between social capital and negative emotions is mediated by social support.
- Hypothesis 3: Social capital is moderated by community sense and social desirability in the relationship with negative emotions.

### 1.4 Importance and significance of the study

This study not only enriches the theoretical research on the relationship between psychosocial factors and negative emotions among the older adult, but also provides scientific evidence for mental health intervention. Through a thorough review of the mechanisms by which social capital, social support, community sense, and social desirability affect negative emotions of the older adult, this research may provide references for government and community decision-makers to formulate effective policies for older adult mental health. Moreover, this study combines the social relationship theory of Marxist philosophy, which stresses the mutualistic relationship between individuals and society, and thus further clarifies the important role of psychosocial factors in the older adult’s mental health.

The Marxist philosophy stresses the significance of social relations in individual development, positing that individual happiness and health are inseparable from the support of the collective and society (27). This theory guided the premise of this study, which argues that the mental health of the older adult not only depends on internal personal factors but is also highly influenced by external social environments and social relationships. In this way, it can effectively improve the mental health status of the older adult by developing their social capital, social support, community sense, and social desirability, thus enhancing their comprehensive development and sense of well-being.

As stated in the “Healthy China 2030 Plan,” an active aging strategy should be promoted to increase the quality of life and mental health of the older adult population. Active aging stresses the all-round development of the older adult in physical, psychological, and social aspects, and encourages the older adult to take part in social activities and increase their social capital and social support (28). This study will provide empirical support for the achievement of the goals of active aging, especially promoting older adult social participation, increasing community sense, and creating social support systems.

In a vast country like China with rapid development, community, as a major source of social support for the older adult, is more critical in its construction and management. The social capital of the older adult can be enhanced, and the occurrence of negative emotions reduced, through enhancing community sense and encouraging social participation and social network support. Mental health intervention measures for the older adult are provided with scientific evidence and practical guidelines for policymakers and community managers to develop more effective measures.

## 2 Methods

### 2.1 Study participants

As a sub-project of the Cardiovascular Disease Screening Program in Dalian, this study explores the association between social psychological factors and negative emotions in the older adult community population. Participants were aged 60–80 years and permanent residents of the communities in Dalian who participated in the cardiovascular disease screening program. Inclusion criteria were: (1) Age 60–80 years; (2) Living in communities within Dalian City; (3) Able to read and write and with at least primary school education; (4) Voluntary participation in the study and signing of informed consent. Exclusion criteria included: (1) Mental illness history; (2) Severe cognitive impairment; (3) Physical illness or other reason precluding completion of the questionnaire. The study was approved by the Ethics Committee of the First Affiliated Hospital of Dalian Medical University [Approval No.PJ-KS-KY-2024-190(X)]. Written informed consent was provided by all participants. A total of 1,000 questionnaires were distributed, and 904 valid responses were received, resulting in an effective response rate of 90.4%. In addition to the routine cardiovascular screening, negative emotions, social capital, social support, sense of community, and social desirability were assessed in participants.

### 2.2 Social capital assessment

The Social Capital Questionnaire was used to assess social capital (13). This questionnaire contains 36 items distributed across 8 dimensions: The 36 items are grouped into local community participation (7 items), proactivity in social contexts (5 items), trust and safety (5 items), neighborhood relations (5 items), family and friends contact (3 items), tolerance for diversity (2 items), life values (2 items), proactivity in a social context (2 items), work connections (3 items), and other(2 items). Since our research is focused on community populations, the majority of older adult individuals in these communities are either retired or not currently in the workforce. Therefore, we excluded the work-related items and used a 33-item version of the questionnaire. The range of total score is 33–132, with higher scores meaning higher social capital. This scale showed good reliability in the study sample with the reliability coefficient (Cronbach’s *α*) of 0.81.

### 2.3 Assessment of negative emotions

Given the characteristics of the older adult community residents and the feasibility of the study, the 10-item version of the Geriatric Depression Scale (GDS-10) was used to assess negative emotions. The 15-item version (GDS-15) is derived from GDS-10 by selecting 10 items that have been shown to have high accuracy and are easier to complete for older adult persons, thus reducing participant burden (29). The score range is 0 to 10, with scores of ≥6 indicating negative emotions and scores of ≤5 indicating no negative emotions. GDS-10 had good internal consistency (Cronbach’s *α* = 0.74).

### 2.4 Social support assessment

The Social Support Rating Scale (SSRS) was used to assess social support. The SSRS was designed to reflect Chinese cultural characteristics and consists of 10 items across three dimensions: There are three types of support: objective support, subjective support, and the utilization of support. The range of total scores is 12–66, with higher scores reflecting greater levels of social support. In Chinese populations, SSRS has been widely used and has good reliability and validity. The reliability of SSRS was excellent as the internal consistency (Cronbach’s *α*) was 0.82.

### 2.5 Sense of community assessment

The Brief Sense of Community Scale (BSCS) was used to assess the Sense of Community, according to the community sense theory (30). BSCS consists of 8 items across four dimensions: The four dimensions of satisfaction with needs (2 items), group membership (2 items), influence within the community (2 items), and emotional connection (2 items). This scale assesses an individual’s sense of belonging, identification, and belief in mutual aid in his/her community. Good psychometric properties have been demonstrated in Chinese populations. The internal consistency (Cronbach’s *α*) of BSCS was 0.80, and it was applicable to older adult community residents.

### 2.6 Social desirability assessment

The Chinese version of the simplified Marlowe-Crowne Social Desirability Scale (MCSDS) was used to assess social desirability (31). The total score range is 0 to 33, with higher scores indicating a stronger tendency to respond in a socially desirable manner. In university students and adult samples in China, the MCSDS has shown good structural and criterion validity. The internal consistency (Cronbach’s *α*) of MCSDS was 0.79, which is sufficient to use MCSDS as a measure of social desirability tendencies in older adult community populations.

### 2.7 Covariates

Age, gender, education level, occupational status, living arrangements, monthly income, number of chronic diseases, and number of medications taken regularly were the covariates included in the analysis. Age was considered as a continuous variable in years. The category of gender was split into male and female. Education level was categorized into three groups: They are junior high school or below, high school/college, and bachelor’s degree or higher. Employed, unemployed, and retired were classified as occupational status. The living arrangements were classified as living alone, living with a spouse, or living with other family members (excluding spouse). Monthly income was categorized into three levels: The price is below 3,000 RMB, 3,000–4,999 RMB, or above 5,000 RMB. The number of chronic diseases and the number of medications taken regularly were classified into three groups: One disease/medication, two diseases/medications, or three or more diseases/medications. Furthermore, continuous variables of individual psychosocial status, such as social capital, social support, sense of community, and social desirability scores were also included in the analysis. Table 1 provides descriptive statistics for all covariates.

**Table 1:** Participant characteristics by negative emotion^a^.

Characteristics	Total	Negative emotion (GDS-15 Score)		p-value*
		No negative emotion	With negative emotion	
		0–5	6–15	
N (%)	904(100)	811(89.71)	93(10.29)	–
Sociodemographic characteristics				
Age (years), mean ± SD	67.46 ± 4.16	67.34 ± 4.11	68.48 ± 4.42	0.027
Gender, *n* (%)				0.146
Female	492 (54.42%)	448 (55.24%)	44 (47.31%)	
Male	412 (45.58%)	363 (44.76%)	49 (52.69%)	
Education level, *n* (%)				0.009
Middle school or below	540 (59.73%)	471 (58.08%)	69 (74.19%)	
Senior secondary/junior college	322 (35.62%)	302 (37.24%)	20 (21.51%)	
University or above	42 (4.65%)	38 (4.69%)	4 (4.30%)	
Occupation				0.688
Employed	82 (9.07%)	73 (9.00%)	9 (9.68%)	
Unemployed	204 (22.57%)	180 (22.19%)	24 (25.81%)	
Retired	618 (68.36%)	558 (68.80%)	60 (64.52%)	
Living arrangement				<0.001
Living alone	125 (13.83%)	96 (11.84%)	29 (31.18%)	
Living with spouse	691 (76.44%)	635 (78.30%)	56 (60.22%)	
Living with other family members (excluding spouse)	88 (9.73%)	80 (9.86%)	8 (8.60%)	
Monthly income (CNY)				0.004
<3,000 CNY	417 (46.13%)	359 (44.27%)	58 (62.37%)	
3,000–4,999 CNY	370 (40.93%)	342 (42.17%)	28 (30.11%)	
≥5,000 CNY	117 (12.94%)	110 (13.56%)	7 (7.53%)	
Number of chronic diseases				0.002
1	185 (20.46%)	170 (20.96%)	15 (16.13%)	
2	578 (63.94%)	526 (64.86%)	52 (55.91%)	
≥3	141 (15.60%)	115 (14.18%)	26 (27.96%)	
Long-term medications count				0.012
1	228 (25.22%)	216 (26.63%)	12 (12.90%)	
2	444 (49.12%)	388 (47.84%)	56 (60.22%)	
≥3	232 (25.66%)	207 (25.52%)	25 (26.88%)	
Psychosocial measures, mean ± SD	3.36 ± 1.65	3.00 ± 1.30	6.53 ± 0.76	<0.001
Social capital	83.57 ± 9.29	84.90 ± 8.60	71.97 ± 6.64	<0.001
Social support	38.30 ± 8.84	38.84 ± 8.93	33.58 ± 6.31	<0.001
Sense of community	24.60 ± 3.19	24.90 ± 2.75	21.91 ± 5.01	<0.001
Social desirability	16.30 ± 3.00	16.40 ± 2.91	15.45 ± 3.63	<0.001

*p-values were calculated using Kruskal-Wallis H test for continuous variables and Fisher’s exact test for categorical variables when expected frequencies were <10. CNY, Chinese Yuan; GDS-15, Geriatric Depression Scale-15; SD, Standard Deviation.

a Continuous variables are presented as mean ± standard deviation; categorical variables are presented as *n* (%).

### 2.8 Statistical analysis

Variables were described as continuous variables using mean ± standard deviation, and categorical variables using frequencies and percentages. Shapiro–Wilk test was used to assess the normality of the variables, with all variables failing to meet the normality assumption (*p* < 0.05). Consequently, comparisons between groups with different levels of depressive symptoms were made using the Kruskal-Wallis H test for continuous variables or chi-square test for categorical variables, with Bonferroni correction where appropriate. Spearman rank correlation analysis was used to examine the relationship between social capital, social support, and depressive symptoms. To address potential multicollinearity concerns among the psychosocial variables, Variance Inflation Factors (VIFs) were calculated for all predictor variables. A VIF threshold of 5 was established as the criterion for problematic multicollinearity. Statistical analyses were carried out using IBM SPSS 29, with a significance level of *α* = 0.05 for two-sided tests.

## 3 Results

### 3.1 Common method biases

**Figure 1:**
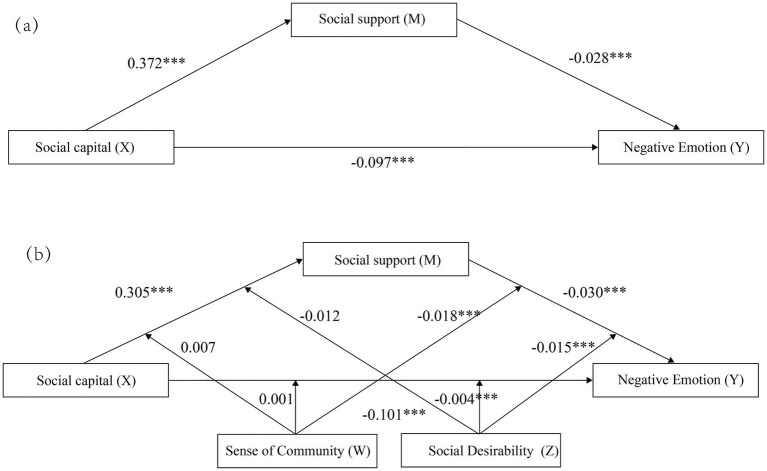
The mediation and moderated mediation models illustrating the relationship between social capital, social support, and negative emotion. **(A)** The initial mediation model: social support as a mediator of the association between Parkinson’s social capital and negative emotion. (Andrew Hayes’s mediation Model 4, ***p < 0.001). **(B)** The final moderated mediation model: The moderated mediation model exploring the role of sense of community and social desirability as moderators. Sense of community and social desirability moderate the pathways in the mediation model between social capital, social support, and negative emotion. (Andrew Hayes’s moderation-mediation Model 76, ***p < 0.001).

To test the mediating effect of social support in the relationship between social capital and depressive symptoms, as well as the moderating effects of sense of community and social desirability, the following models were constructed: (1) Model 4 (simple mediation model): Depressive symptoms as the dependent variable, social capital as the independent variable, and social support as the mediating variable. (2) Model 76 (dual moderation mediation model): Model 4 was expanded to include the sense of community and social desirability as moderating variables, to examine their effects on the direct and indirect (via social support) relationships between social capital and depressive symptoms (Figure 1). Since all variable data in this study were collected from participants’ self-reports, common method bias may be a problem. We assessed common method bias by performing Harman’s single factor test using confirmatory factor analysis (CFA). Fit indices for the single factor model were poor (χ^2^/df = 5.26, RMSEA = 0.092, CFI = 0.811, TLI = 0.785), and factor loadings ranged from 0.132 to 0.907 and residual variances were substantial. The results of these analyses suggest that no single factor explains the majority of the variance, and thus common method bias is unlikely to have a large impact on our findings.

### 3.2 The participants’ characteristics

Among the 904 participants, the distribution of negative emotions severity based on GDS-15 scores was: 89.71% (n = 811) no negative emotions and 10.29% (n = 93) mild negative emotions. Significant differences across negative emotion levels were seen in several domains (Table 1). Those with higher negative emotion levels had lower educational attainment (mainly below middle school, *p* = 0.009), were more likely to live alone (*p* < 0.001), and reported lower monthly income (*p* = 0.004), with 62.37% of mildly negative emotional persons having monthly income below 3,000 CNY, compared to 44.27% in the non-negative emotional group. In addition, the negative emotional group had fewer chronic diseases and long-term medication types (*p* < 0.05). Significant negative correlations were found between negative emotion severity and all psychosocial measures (*p* < 0.001), with lower scores on social capital, social support, sense of community, and social desirability in the negative emotional group.

### 3.3 Correlation analysis

The study variables were found to be significantly associated with each other in bivariate correlation analysis (Table 2). Social capital was strongly negatively correlated with negative emotions (*r* = −0.643,*p* < 0.001), and the two were closely related, but further exploration of their causal relationship and the underlying mechanisms is needed. Negative correlations were also found between negative emotions and social support: social support correlated with negative emotions at the levels of *r* = −0.361 (*p* < 0.001), sense of community: social support correlated with negative emotions at the levels of *r* = −0.141 (p < 0.001), and social desirability: social support correlated with negative emotions at the levels of *r* = −0.082 (*p* < 0.05), implying that a sound social support system and positive community environment can moderate the risk of negative emotions for older adults. Along with this, social capital was correlated in positive direction with social support (*r* = 0.426, *p* < 0.001), sense of community (*r* = 0.180, *p* < 0.001) and with social desirability (*r* = 0.092, *p* < 0.01); presumably, these revealed mutual reinforcement. The preliminary correlation patterns are used as a basis for further exploration of the complex relationships between negative emotions and psychosocial factors.

**Table 2:** Correlations for the main variables.

Variable	1	2	3	4	5
1. Social capital	–				
2. Negative emotion	−0.643***	–			
3. Social support	0.426***	−0.361***	–		
4. Sense of community	0.180***	−0.141***	0.348***	–	
5. Social desirability	0.092**	−0.082*	0.223***	0.590***	–

***P < 0.001, **P < 0.01, *P < 0.05.

### 3.4 Multiple regression analysis

Collinearity diagnostics revealed no significant multicollinearity issues among the predictor variables, with all VIF values below 2 (see Supplementary Tables S1, S2), confirming the statistical independence of the social capital, social support, community sense, and social desirability measures. The regression model accounted for 34.2% of the total variance in social support (R^2^ = 0.342, adjusted R^2^ = 0.334, *F* = 42.231, *p* < 0.001). Social capital (*β* = 0.311, *p* < 0.001) and sense of community (*β* = 0.423, *p* < 0.001) were significant predictors, and living arrangements (*β* = 0.082, *p* = 0.003) was a relatively weak but significant predictor. No significant associations were found with other sociodemographic factors or with social desirability (Supplementary Table S1). Regarding negative mood, the model explained 43.4% of the variance (R^2^ = 0.434, adjusted R^2^ = 0.426, *F* = 56.867, *p* < 0.001). The strongest association was found for social capital (*β* = −0.609, *p* < 0.001), followed by living arrangements (*β* = −0.069, *p* = 0.007) and age (*β* = 0.064, *p* = 0.014). Marginally significant association was found with social support (*β* = −0.059, *p* = 0.058). No significant associations were found with other sociodemographic factors, sense of community, or social desirability (Supplementary Table S2). After these regression analyses, mediation analysis using PROCESS Model 4 was conducted to test the mediating role of social support in the relationship between social capital and negative emotions.

### 3.5 Social support as mediating effect

The mediating role of social support in the relationship between social capital and negative emotions was examined with mediation analysis using PROCESS Model 4 (Table 3). Results showed that social capital was significantly associated with social support [*B* = 0.372, 95% CI] Social support was negatively associated with negative emotions [*B* = −0.028, 95% CI: (−0.034, −0.022)], and physical activity [*B* = 0.315, 95% CI: (0.230, 0.400)] and healthy eating [*B* = 0.430, 95% CI: (0.320, 0.540)] were positively associated with negative emotions. The direct effect of social capital on negative emotions remained significant [*B* = −0.097, 95% CI]: the direct effect of social support on psychological distress was significant [*B* = −0.088, 95% CI: (−0.105, −0.088)], and the indirect effect through social support was also significant [*B* = −0.015, 95% CI: (−0.016, −0.013)]. The total effect was −0.111 95% CI: The indirect effect was 0.013 [95% CI (−0.120, −0.103)], and accounted for 13.51% of the total effect, suggesting that social support partially mediated the relationship between social capital and negative emotions in community-dwelling older adults.

**Table 3:** Testing the mediating effect of social support on Negative emotion.

Variable	Path	B	SE	LLCI	ULCI
Total effect	Social capital (X) – Negative emotion (Y)	−0.111	0.005	−0.120	−0.103
Direct effect	Social capital (X) – Social support (M)	0.372	0.029	0.315	0.430
	Social support (M) – Negative emotion (Y)	−0.028	0.005	−0.034	−0.022
	Social capital (X) – Negative emotion (Y)	−0.097	0.004	−0.105	−0.088
Indirect effect	Social capital (X)–Social support (M) – Negative emotion (Y)	−0.015	0.001	−0.016	−0.013

B, unstandardized coefficients; SE, the standard error of indirect effects estimated; LLCI, lower limit confidence interval; ULCI, upper limit confidence interval.

### 3.6 Moderated mediation analysis

**Figure 2:**
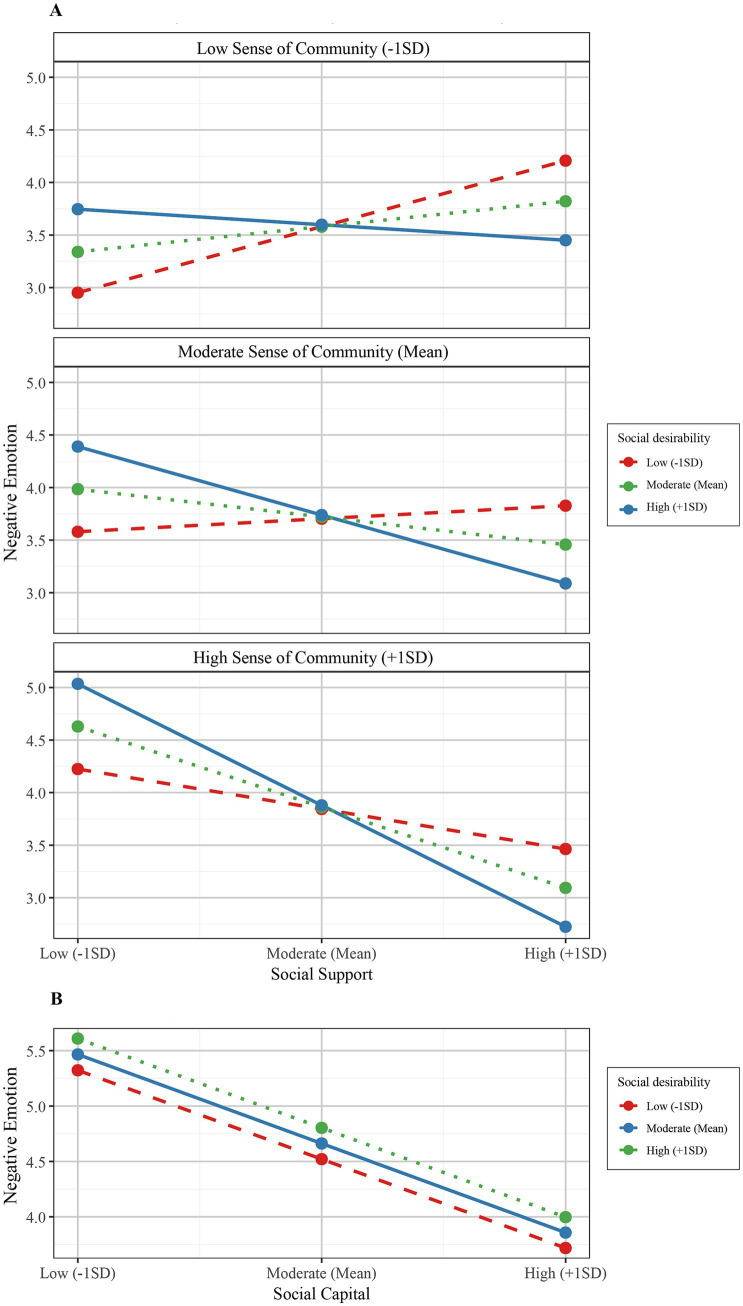
Moderating effects of sense of community and social desirability on the relationship between social support and negative emotion **(A)** and the direct effect of social capital on negative emotion **(B)**. **(A)** Effects of social support on negative emotion moderated by sense of community and social desirability. **(B)** Direct effect of social capital on negative emotion moderated by social desirability. Note: Panel A shows how the relationship between social support and negative emotion varies across different levels of sense of community (low: -1SD; moderate: Mean; high: +1SD) and is moderated by levels of social desirability (low, moderate, high). Panel B illustrates the moderating effect of social desirability levels (low, moderate, high) on the direct relationship between social capital and negative emotion.

In initial exploration with PROCESS Model 76, the moderating roles of sense of community (SOC) and social desirability (SD) in the relationship between social capital and negative emotions through social support were examined (Supplementary Table S3). The analysis revealed that SOC significantly moderated the relationship between social support and negative emotions [MW: A significant main effect was found for SOC (W: *β* = 0.044, *p* < 0.001)], as well as for β (W: *β* = −0.018, *p* < 0.001). Similarly, SD demonstrated significant interaction with social support (MZ: However, its main effect was not significant) (Z: *β* = 0.006, *p* = 0.531), although its interaction was (*β* = −0.015, *p* < 0.001). Notably, SD’s moderation of the direct path from social capital to negative emotions, while statistically significant [X*Z: A minimal effect size (R^2^-change = 0.003)] was found for the effect of the number of years of experience (*β* = −0.004, *p* < 0.001). Conditional effects analysis showed systematic variation in the relation between social support and negative emotions by levels of both moderators (Table 4). The effect of social support on negative emotions was positive when SOC was low (−1SD), but became slightly negative as SD increased (*β* ranging from 0.071 to −0.017). This relationship became consistently negative at mean levels of SOC, with stronger effects at higher levels of SD (*β* ranging from 0.014 to −0.074). The highest levels of SOC (+1SD) were associated with the strongest negative associations, particularly when combined with high SD (β from −0.043 to −0.131). Figure 2A shows that the protective effect of social support against negative emotions was strongest when both moderators were high (*β* = −0.131, *p* < 0.001), and reversed when both moderators were low (*β* = 0.071, *p* < 0.001). These findings were further supported by the conditional indirect effects analysis, where the mediating effect of social support was significantly different across different combinations of moderators, as indicated by significant indices of conditional indirect effects with confidence intervals not including zero. As shown in Figure 2B, although SD’s moderation of the direct social capital-negative emotions pathway reached statistical significance, the conditional effects across different SD levels showed minimal variation low: This further supports that SD primarily influences through moderating the mediating pathway of social support rather than the direct pathway (moderate: *β* = −0.087; high: *β* = −0.088; *β* = −0.086).

**Table 4:** Conditional indirect effects of social capital on negative emotion through social support at different levels of sense of community and social approval.

Sense of community (W)	Social desirability (Z)	B	SE	LLCI	ULCI
Low (−1SD)	Low (−1SD)	0.071	0.004	0.063	0.079
	Moderate (Mean)	0.027	0.004	0.019	0.035
	High (+1SD)	−0.017	0.006	−0.029	−0.004
Moderate (Mean)	Low (−1SD)	0.014	0.005	0.005	0.023
	Moderate (Mean)	−0.030	0.003	−0.036	−0.024
	High (+1SD)	−0.074	0.005	−0.083	−0.064
High (+1SD)	Low (−1SD)	−0.043	0.006	−0.055	−0.031
	Moderate (Mean)	−0.087	0.004	−0.095	−0.078
	High (+1SD)	−0.131	0.005	−0.139	−0.122

B, unstandardized coefficients; SE, the standard error of indirect effects estimated; LLCI, lower limit confidence interval; ULCI, upper limit confidence interval; SD, standard deviation.

## 4 Discussion

### 4.1 Main findings and analysis

The main findings of this study are that social capital, social support, community sense, and social desirability have significant effects on negative emotions of the older adult, and there are complicated interactions among these factors. Our analysis revealed significant associations between the study variables (*r* = −0.643, *p* < 0.001). Furthermore, social support plays a significant mediating role between social capital and negative emotions (*B* = −0.015, 95% CI: [−0.016, −0.013]), and community sense and social desirability are moderating variables that significantly moderate the relationship between social capital and social support with negative emotions (*p* < 0.001). In particular, negative effects of social capital and social support on negative emotions are more pronounced when there is a higher community sense and social desirability. Moreover, these findings support and offer new insights on how to interpret mental health in the older adult (14).

### 4.2 The mechanism of social support

Social support, as a result of social capital, is found to mediate the effect of social capital on negative emotions, suggesting that it is a core mediating variable in reducing negative emotions. This is consistent with previous social support buffering theory (14). Additionally, social support will aid in an increase in an individual’s social participation and sense of belonging, thus enhancing their mental health. As a result, enhancing the level of social support among the older adult is an important strategy for prevention and intervention in negative emotions. The traditional Chinese culture sources its social support from family and community. According to Confucianism, “benevolent” and “harmonious,” family relations involve mutual support between family members as well as a collectivist spirit in the community. In the meantime, social support under this cultural background is not only emotional comfort but also reflects social norms and values. The incidence of negative emotions among the older adult in China is significantly decreased by social support from family and community (12). The result suggests that the role of social support in the mental health of older adult Chinese people is culturally specific and should be understood and utilized within the cultural context.

### 4.3 Moderating effects of community sense and social desirability

The study also revealed that community sense and social desirability, as moderating variables, have important effects on the relationship between social capital and negative emotions. In particular, the negative effect of social capital on negative emotions can be greatly enhanced by higher community sense and active social participation. This finding is consistent with previous community sense theory, which postulates that health relies on the sense of belonging, participation, and identification, and that these conditions create among belonging individuals (32). Under these conditions, enhancing community sense and social participation will promote sense identification and belonging to the collective, thereby lowering the occurrence of negative emotions. In addition to that, social participation can further provide more social support; participation in collective activities and interaction with others enhance an individual’s social capital and community sense, which are also associated with fewer negative emotions. In addition, the study revealed that the moderating effects of community sense and social desirability are present at different levels of social capital and their effect on negative emotions. In particular, the negative effect of social capital on negative emotions is exacerbated under conditions of high community sense and active social participation. This indicates that community sense, along with social participation, may enhance the beneficial consequences from social capital on mental health (17). The results provide further support to social capital theory and community sense theory in that social interaction and community participation are important factors in the mental health of the older adult (32).

Our finding that community sense and social desirability jointly moderate the relationship between social support and negative emotions aligns with Marx’s dialectical materialist understanding of human development. Marx posited that individual well-being emerges from the dialectical interaction between objective social conditions and subjective consciousness (33). In our study, community sense represents the objective dimension of social integration (reflecting material conditions), while social desirability captures subjective normative perceptions (reflecting social consciousness). Their interactive effect demonstrates how these dialectical components work together to shape emotional experiences.

### 4.4 The role of social desirability

In addition, social desirability, an intrinsic social incentive factor, also plays a moderating role in the relationship between social support and negative emotions in this study. Negative emotions are alleviated by social desirability because it increases individuals’ self-worth and social identity. This finding is consistent with self-actualizing theory from humanistic psychology that advocates for the use of self-actualizing and positive reinforcement in society to affect people’s mental health (34). Social desirability in Chinese social culture is not only reflected in the desire for others to recognize but also in the process of individuals realizing self-worth through social roles and social status (20). By receiving high social desirability, an older adult individual can increase his or her self-esteem and social identity and therefore will not develop negative emotions (35). However, excessive social desirability may force individuals to stifle their true emotions to win the approval of others, bringing psychological stress, which eventually may lead to negative emotions (19, 36). Thus, social desirability serves a dual purpose in enhancing and reducing negative emotions, and practical applications must balance both aspects.

### 4.5 Comparison with previous studies

This research further validates the roles of social support and social capital in negative emotions of the older adult and is the first to explore the moderating roles of community sense and social desirability. Besides enriching theoretical research in related fields, these findings provide new perspectives for the practical prevention and intervention of negative emotions among the older adult. For instance, the negative emotions are remarkably mitigated in older people by social support, particularly in areas where community support has been firmly established (37). This study also verifies the function of social support in minimizing the occurrence of negative emotions in the older adult (37). Additionally, the social capital has a positive effect on mental health by increasing social support (38). This study also shows that the negative correlation between social capital and negative emotions is supported in the older adult population (38). Furthermore, the community sense has a critical impact on increased social support networks and psychological resilience (32). Research on social desirability also provides significant support for the positive effect of social desirability on individual mental health, consistent with the findings on social desirability in this study (39). More importantly, this study is the first to examine the moderating effect of social desirability in the relationship between social support and negative emotions, which extends research perspectives in the existing literature. Most previous studies have focused on the direct effects of social support and social capital on mental health, with little attention paid to the moderating role of intrinsic social incentive factors such as social desirability. Consequently, the results of this study not only confirm existing theories but also suggest new research directions for future studies.

### 4.6 Significance for China’s national conditions

This study provides empirical support for the achievement of active aging goals, especially encouraging social participation, increasing community sense, and constructing social support systems for the older adult. As a vital source of social support for the older adult, the community is of special importance in the construction and management of communities in the context of China’s rapid development. Increasing community sense and social participation not only enhance the social capital of the older adult but also decrease the incidence of negative emotions by strengthening social support networks. Scientific evidence and practical guidelines for policymakers and community managers to formulate more effective mental health intervention measures for the older adult are provided (40).

### 4.7 Policy recommendations and practical applications

According to the findings of this study, governments, communities, and other related decision-makers should build social support systems for the older adult. Specifically, policymakers can enhance social support and social capital among the older adult through the following measures:

#### 4.7.1 Enhancing community service facilities

Improve community-based services for the older adult, including senior activity centers, psychological counseling rooms, and medical service stations, to provide diverse services that meet the needs of the older adult. Globally developed community service facilities can offer not only the basic materials essential for survival but also platforms for social and emotional support.

#### 4.7.2 Promoting older adult social participation

Organize and support community activities in which the older adult can participate on a volunteering basis, join interest groups, or attend health lectures to promote social interaction and a feeling of belonging. Social participation helps the older adult establish new social relations, enhance social capital, and reduce the occurrence of negative emotions.

#### 4.7.3 Enhancing community sense construction

Through community construction projects, improve the older adult’s sense of identification and belonging to the community, create a harmonious community atmosphere, and enhance individuals’ sense of community responsibility. Improving community sense not only helps the older adult maintain their social support networks but also aids in sustaining their mental health and well-being.

#### 4.7.4 Elevating social desirability levels

Employ publicity and educational activities to encourage societal recognition and approval of the older adult, thereby increasing their self-esteem and social identity. Enhancing the older adult’s sense of self-worth through social desirability will encourage them to actively participate in community activities and reduce negative emotions. It is necessary for community decision-makers to pay attention to the older adult’s social capital, especially distinguishing between rural and urban older adult social capital, and to adopt differentiated intervention strategies to improve the social capital of the older adult. In rural areas, strengthening neighborhood relationships and family support can build social capital, while in urban areas, increasing community participation and diversifying social activities can enhance social capital. To prevent and alleviate the older adult’s negative emotions, social workers and mental health professionals should actively participate in mental health interventions for the older adult, offering professional psychological counseling and support services to enable the older adult to build and maintain strong social support networks.

### 4.8 Limitations and future directions

However, this study has several limitations despite achieving some findings. Firstly, this study employed a cross-sectional design, which limits our ability to establish temporal sequences among variables. Although our theoretical framework posits that social capital influences negative emotions through social support, we cannot exclude the possibility of reverse causality, wherein negative emotions might affect individuals’ capacity to access social capital and support. We acknowledge that without longitudinal data, the mediation analysis in this study can only reflect associational patterns between variables rather than strict causal chains. Therefore, our findings should be interpreted as correlational rather than causal evidence. Future research should employ longitudinal designs to verify the temporal sequence and causal relationships among these variables. Furthermore, the older adult population was used exclusively in this study; future studies should address other age groups to examine the practical validity of these psychosocial factors in the development of negative emotions. Secondly, because this study conducted an in-depth survey of the older adult population in communities of Dalian City, caution is needed in generalizing the results. The impact of social capital and community sense on negative emotions may differ due to various social and cultural backgrounds and levels of economic development in different regions. Future work should therefore conduct studies in different regions and cultural contexts to increase the breadth of applicability of the results. Additionally, the data in this study were collected primarily via self-report questionnaires, which may contribute to self-report bias and/or social desirability bias. Harman’s single factor test was used to check for common method bias; however, future research using multiple data collection methods, such as interviews and observations, could enhance the authenticity and reliability of the data.

## 5 Conclusion

By surveying the older adult population in the communities of Dalian City, this study found that social capital, social support, community sense, and social desirability have a significant impact on negative emotions of the older adult and investigated the complicated relationships among these psychosocial factors. The results show that strengthening community sense can enhance social capital and social support, and prevent and alleviate negative emotions in the older adult. Additionally, social desirability, an intrinsic social incentive factor, plays an important role in promoting the mental health of the older adult. In addition to providing new theoretical perspectives for academia, these findings also provide empirical support for government and community decision-makers to formulate mental health intervention measures for the older adult. With China’s population aging and rapid social development, improving the levels of social capital and social support of the older adult, encouraging the older adult to actively participate in community activities, improving community sense and social desirability, will have profound impacts on the improvement of the quality of life and mental health of the older adult.

## Data Availability

The raw data supporting the conclusions of this article will be made available by the authors, without undue reservation.
