# Supplementary material for: The hidden network: community sense, social desirability, and their protective influence on negative emotions in aging populations
Source: Front Public Health. 2025 Apr 23;13:1572044. doi: 10.3389/fpubh.2025.1572044 (PMC12055499; doi:10.3389/fpubh.2025.1572044)
Supplement: Supplementary file 1 [file Supplementary_file_1.docx]

**Appendix A. Supplementary data**

The Hidden Network: Community Sense, Social Desirability, and Their Protective Influence on Negative Emotions in Aging Populations

**Supplementary Information**

- **Table S1**. Multiple regression analysis of factors associated with social support among community-dwelling older adults.
- **Table S2.** Multiple regression analysis of factors associated with negative emotion among community-dwelling older adults.
- **Table S3.** Testing the moderated mediating effect of social capital on negative emotion by social support, sense of community and social approval.

| **Table S1.** Multiple regression analysis of factors associated with social support among community-dwelling older adults. | | | | | | | | | | |
| --- | --- | --- | --- | --- | --- | --- | --- | --- | --- | --- |
|  | **Unstandardized coefficient** |  | **SE** | **Standardized coefficient** |  | **t** | **p** | **Collinear statistics** |  | **VIF** |
|  | **B** |  |  | **β** |  |  |  | **Tolerance** |  |  |
| Constant | -13.134 |  | 4.976 |  |  | -2.640 | 0.008 |  |  |  |
| Age | -0.061 |  | 0.059 | -0.029 |  | -1.038 | 0.299 | 0.955 |  | 1.048 |
| Gender | -0.568 |  | 0.486 | -0.032 |  | -1.169 | 0.243 | 0.984 |  | 1.016 |
| Education level | 0.198 |  | 0.414 | 0.013 |  | 0.477 | 0.634 | 0.986 |  | 1.015 |
| Occupation | -0.040 |  | 0.373 | -0.003 |  | -0.108 | 0.914 | 0.977 |  | 1.024 |
| Living arrangement | 1.489 |  | 0.498 | 0.082 |  | 2.989 | 0.003 | 0.991 |  | 1.009 |
| Monthly income | -0.139 |  | 0.349 | -0.011 |  | -0.398 | 0.691 | 0.984 |  | 1.016 |
| Number of Chronic Diseases | -0.193 |  | 0.412 | -0.013 |  | -0.467 | 0.640 | 0.945 |  | 1.058 |
| Long-term Medications Count | -0.214 |  | 0.339 | -0.017 |  | -0.632 | 0.528 | 0.985 |  | 1.016 |
| Social Capital | 0.295 |  | 0.027 | 0.311 |  | 11.100 | <0.001 | 0.942 |  | 1.062 |
| Sense of Community | 1.175 |  | 0.093 | 0.423 |  | 12.692 | <0.001 | 0.663 |  | 1.509 |
| Social Desirability | 0.011 |  | 0.097 | 0.004 |  | 0.115 | 0.909 | 0.678 |  | 1.475 |
| R^2^ = 0.342, adjusted R^2^ = 0.334, F = 42.231, *p* < 0.001. | | | | | | | | | | |

| **Table S2.** Multiple regression analysis of factors associated with negative emotion among community-dwelling older adults. | | | | | | | | | | |
| --- | --- | --- | --- | --- | --- | --- | --- | --- | --- | --- |
|  | **Unstandardized coefficient** |  | **SE** | **Standardized coefficient** |  | **t** | **p** | **Collinear statistics** |  | **VIF** |
|  | **B** |  |  | **β** |  |  |  | **Tolerance** |  |  |
| Constant | 11.165 |  | 0.866 |  |  | 12.900 | <0.001 |  |  |  |
| Age | 0.025 |  | 0.010 | 0.064 |  | 2.467 | 0.014 | 0.953 |  | 1.049 |
| Gender | 0.125 |  | 0.084 | 0.038 |  | 1.487 | 0.137 | 0.982 |  | 1.018 |
| Education level | -0.085 |  | 0.072 | -0.030 |  | -1.189 | 0.235 | 0.985 |  | 1.015 |
| Occupation | -0.003 |  | 0.065 | -0.001 |  | -0.046 | 0.963 | 0.977 |  | 1.024 |
| Living arrangement | -0.234 |  | 0.087 | -0.069 |  | -2.692 | 0.007 | 0.981 |  | 1.019 |
| Monthly income | -0.103 |  | 0.060 | -0.043 |  | -1.697 | 0.090 | 0.984 |  | 1.017 |
| Number of Chronic Diseases | 0.117 |  | 0.071 | 0.042 |  | 1.634 | 0.103 | 0.945 |  | 1.058 |
| Long-term Medications Count | 0.046 |  | 0.059 | 0.020 |  | 0.789 | 0.430 | 0.984 |  | 1.016 |
| Social Capital | -0.108 |  | 0.005 | -0.609 |  | -21.962 | <0.001 | 0.828 |  | 1.208 |
| Social Support | -0.011 |  | 0.006 | -0.059 |  | -1.896 | 0.058 | 0.658 |  | 1.521 |
| Sense of Community | 0.007 |  | 0.017 | 0.014 |  | 0.423 | 0.672 | 0.561 |  | 1.781 |
| Social Desirability | 0.009 |  | 0.017 | 0.016 |  | 0.533 | 0.594 | 0.678 |  | 1.475 |
| R^2^ = 0.434, adjusted R^2^ = 0.426, F = 56.867, *p* < 0.001. | | | | | | | | | | |

|  |  |  |  |  |  |  |  |  |  |  |  |
| --- | --- | --- | --- | --- | --- | --- | --- | --- | --- | --- | --- |
| **Table S3.** Testing the moderated mediating effect of social capital on negative emotion by social support, sense of community and social approval. | | | | | | | | | | | |
|  | **Social support (M)** | | | | |  | **Negative emotion (Y)** | | | | |
|  | **B** | **SE** | ***P-value*** | **LLCI** | **ULCI** |  | **B** | **SE** | ***P*-value** | **LLCI** | **ULCI** |
| Social capital (X) | 0.305 | 0.026 | <0.001 | 0.253 | 0.357 |  | -0.101 | 0.003 | <0.001 | -0.107 | -0.096 |
| Social support (M) | - | - | - | - | - |  | -0.030 | 0.003 | <0.001 | -0.036 | -0.024 |
| Sense of Community (W) | 1.174 | 0.093 | <0.001 | 0.991 | 1.356 |  | 0.044 | 0.010 | <0.001 | 0.025 | 0.063 |
| Social Approval (Z) | 0.025 | 0.097 | 0.800 | -0.166 | 0.216 |  | 0.006 | 0.009 | 0.531 | -0.013 | 0.024 |
| X*W | 0.007 | 0.010 | 0.503 | -0.013 | 0.027 |  | 0.001 | 0.001 | 0.528 | -0.001 | 0.003 |
| X*Z | -0.012 | 0.012 | 0.316 | -0.034 | 0.011 |  | -0.004 | 0.001 | <0.001 | -0.007 | -0.002 |
| M*W | - | - | - | - | - |  | -0.018 | 0.001 | <0.001 | -0.020 | -0.016 |
| M*Z | - | - | - | - | - |  | -0.015 | 0.001 | <0.001 | -0.017 | -0.012 |
| Abbreviations: B = unstandardized coefficients; SE = the standard error of indirect effects estimated; LLCI = lower limit confidence interval; ULCI = upper limit confidence interval. | | | | | | | | | | | |
